# Wisdom on the 3 Cs—Compassion, Culture, and Courage—from One of My Heroes, President Jimmy Carter

**DOI:** 10.4269/ajtmh.21-1147

**Published:** 2022-01-05

**Authors:** Julie Jacobson

**Affiliations:** Managing Partner and Co-Founder Bridges to Development 2021 ASTMH President E-mail: jjacobson@bridgestodevelopment.org

It was my first trip to Nigeria in 2007. The trip hadn’t gone easily, with lots of bumps in the road. I arrived completely exhausted, not knowing exactly where I was besides a hotel in Abuja. I left my room realizing I was starving and went downstairs to find the restaurant. I walked across an outdoor corridor courtyard and opened the door to an Italian restaurant. It was dark inside. I looked around trying to see if a table was free, to see that it actually was mostly empty. As I looked around, I heard my name, “Hey Julie, come on over and join us.” To my utter surprise it was President Jimmy Carter. I was in Abuja to attend a Carter Center meeting, but I did not realize that President Carter was going to be there himself! He was having dinner with his team and family the night before the meeting started. President Carter generously beckoned me over to join them, demonstrating his level of engagement with his team and his commitment to working to control, eliminate, and eradicate neglected tropical diseases.

This commitment and personal attention to be there, hear the truth, and see what is happening himself, is part of the reason for President Carter’s and The Carter Center’s success in global health. Honor the people and the partners, work in partnership, knowing they have a wisdom and understanding that you will never have. When I think of courage, compassion and culture - the three Cs that framed my year as ASTMH President - I can think of few people that exemplify each of those characteristics so fully as President Carter. During that meal that I remember fondly, I heard so many stories. There were stories of China behind the curtain, backroom conversations, and amazing adventures. I was so tired I don’t remember details, except for a strong feeling of warmth, of acceptance, and a profound sense of privilege to be included in the conversation.

I’ve met President Carter several times and always had that sense of warmth and welcoming. I had the honor to interview President Carter and Bill Gates Sr. at the Bill & Melinda Gates Foundation for a staff event. It was February 2012, I had just returned from the signing of the London Declaration. It was an amazing moment. Bill Sr. and President Carter have had a beautiful friendship over many years, sharing life experiences and a desire to make the world a better place, and not turning away from the difficult challenges or the barriers to solve them. I’ve now had the privilege to interview President Carter a second time, this time around the topic of compassion, culture, and courage.

**Thank you, President Carter, for taking the time to share some of your experience with the ASTMH family and beyond. I remember the first time I interviewed you, in February 2012. I had the opportunity to interview you and Bill Gates Sr. at the Bill & Melinda Gates Foundation. It was a huge moment for me. I know you and Bill Gates Sr. were friends, and you two are both very compassionate human beings. Would you please share a little bit about your friendship with Bill Gates Sr. and any reflections on that friendship and compassion?**

Thank you for inviting me to participate, and congratulations on your presidency of the American Society of Tropical Medicine and Hygiene (ASTMH). I was fortunate to spend time with Bill Gates Sr. over the years, including a trip in 2002 to several countries in Africa. Bill was traveling as cochair of the Gates Foundation, together with his wife, Mimi, and we visited some of the health clinics receiving humanitarian aid to treat HIV/AIDS. Bill warmly and personally greeted patients. I always appreciated our times together because we both believed that dignity is a human right. From that perspective, most wounds can be healed.

**Figure:**
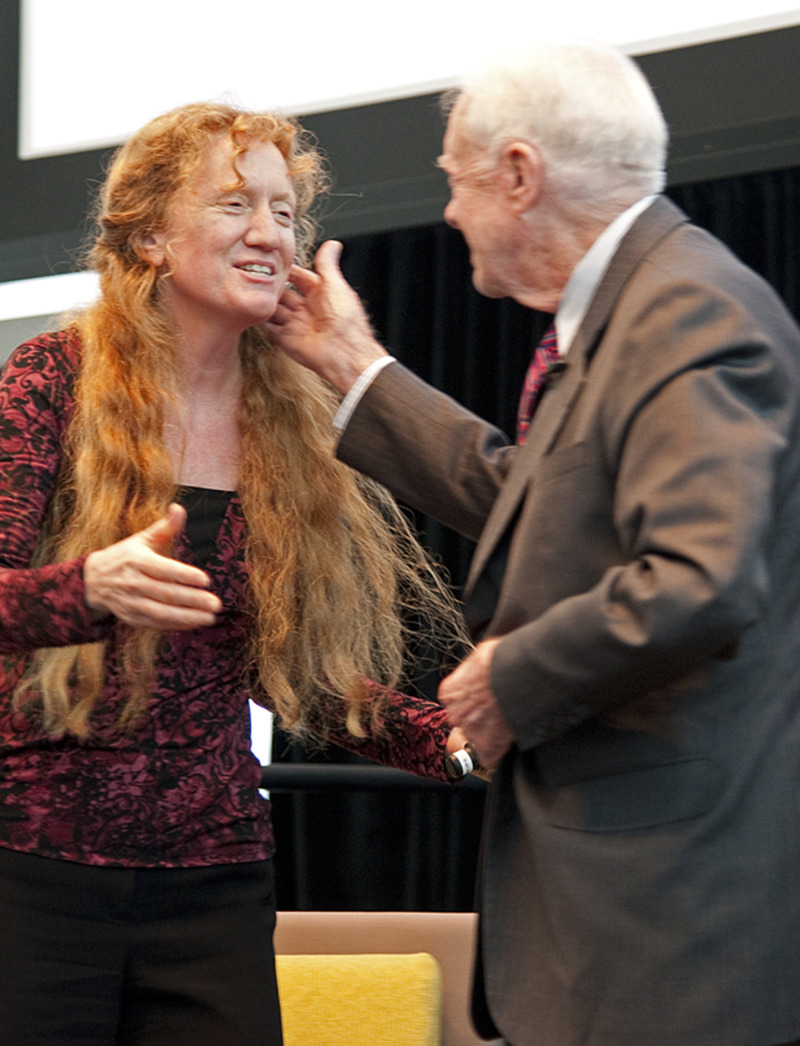
2021 ASTMH President Julie Jacobson, MD, DTM&H, FASTMH with former US President Jimmy Carter, February 2, 2012.

**The 2021 theme at the ASTMH is Compassion, Culture, and Courage. It’s important for us to nurture these attributes as we face current challenges. I see courage in standing up for what one believes in and the use of one’s voice. You have used your voice to stand up for principles that you believe in throughout your life. Where do you find the courage to speak up? Could you describe a time when you had to find a way to generate the courage to do what was right?**

I grew up in the segregated South. When I went with my best friend—a Black child—to Americus to watch a movie, we had to ride in separate train cars, and it upset me. Later, I would come to understand that feeling to be injustice, and it shaped my dedication to civil rights. In the beginning of my political career, I lost many votes, and even my first bid for governor, because I voiced my belief in equality. It is not always easy to stand by your beliefs. However, I have deep faith in civil and human rights. I often recall my high school teacher, Miss Julia Coleman, telling us, “We must adjust to changing times and still hold to unchanging principles.” Through each stage of my life, I have drawn courage from those words.

**Culture is rich, complex, and fascinating. You have seen so much of the world as a political leader and humanitarian. I’m curious about the role of culture in promoting peace. How do you find the appropriate balance between honoring cultural beliefs and finding the way to ensure peace?**

I am fortunate to have had the privilege of participating in hundreds of historical, cultural, and social experiences throughout the world, and I have learned from all of them. I have built lasting relationships with people in some of the most remote places you can imagine. I have found that despite the diversity of our cultures, we all relate through a common foundational desire for peace and equality.

The bridge between cultural differences lies in a shared desire for peace and opportunity, as evidenced by effective cease-fires in various countries over the years. The longest humanitarian cease-fire in history was in Sudan in 1995 to deliver public health services, including for the Guinea Worm Eradication Program. Violence is an unfortunate part of life, but ultimately, everyone wants the same things: dignity and security.

**How has compassion played a role in your work? How has it shaped which issues you choose to take on?**

My life has been guided by the basic principles of compassion, respect, and understanding. Observing injustice can be overwhelming. Global suffering is more than any one person can endure; still, I can attest to the healing power of empathy and compassion.

One of the most moving experiences was a trip with Rosalynn to Ghana. We visited a number of communities afflicted with Guinea worm, which was affecting not just comfort but the ability to farm or contribute to the household. We saw villages of people in unnecessary pain from a disease that doesn’t exist in developed nations. The inequity I witnessed was influential in my dedication to eradicate that awful disease, but also because it highlighted the power that compassion can have in changing lives.

Regardless of differences, people will improve their own lives when provided with the necessary skills, knowledge, and access to resources. This belief is one of the Carter Center’s guiding principles.

**What would you like to share with the diverse members of the ASTMH from around the world working to overcome the burden of tropical diseases and support a healthier, more resilient world? Any advice on the way forward?**

For almost four decades, The Carter Center has been dedicated to collaborative action to address the most difficult issues facing our world. ASTMH and its members embody these same principles, working together to address neglected tropical diseases in the most endemic regions worldwide. I am encouraged by the power of a group of dedicated individuals united around the promotion of peace and health. ASTMH is doing important work with local governments and ministries of health to provide resources and to share and deploy innovative solutions. The commitment of medical and scientific professionals working toward a common humanitarian goal inspires me.

Thank you, President Carter, for your time. Thank you for your words of wisdom. Thank you also for your commitment to global health and tropical medicine. We at ASTMH continue to learn with you and from you. You’re an inspiration to us all, and we look forward to continuing to learn with you and from you as we address the challenges ahead. Thank you for sharing your experience to guide us and inspire us on that path.

